# Supplementary material for: Facilitating population genomics of non-model organisms through optimized experimental design for reduced representation sequencing
Source: BMC Genomics. 2021 Aug 21;22:625. doi: 10.1186/s12864-021-07917-3 (PMC8380342; doi:10.1186/s12864-021-07917-3)
Supplement: Supplementary file 1 — Additional file 1. Samples used for reduced representation sequencing (RRS) optimization. DNA from these samples was used for empirical restriction enzyme digestions with different enzymes (single digest EcoRI, PstI, MspI, or double digest EcoRI-MspI) and for RRS pilot libraries. Some samples were extracted twice as replicates (marked as _rep in sample ID). Three samples per species (family in the case of ostracods) were used for empirical digestions. The amphipod (C. obesa and E. pontomedon) samples and one T. loennbergii were used for empirical digestions, but not included in any RRS library. [file 12864_2021_7917_MOESM1_ESM.docx]

Supplemental Information for:

**Facilitating population genomics of non-model organisms through optimized experimental design for reduced representation sequencing**

Henrik Christiansen^1*^, Franz M. Heindler^1^, Bart Hellemans^1^, Quentin Jossart^2^, Francesca Pasotti^3^, Henri Robert^4^, Marie Verheye^4^, Bruno Danis^5^, Marc Kochzius^2^, Frederik Leliaert^3,6^, Camille Moreau^5,7^, Tasnim Patel^4^, Anton P. Van de Putte^1,4,5^, Ann Vanreusel^3^, Filip A. M. Volckaert^1^ & Isa Schön^4^

^1^ KU Leuven, Laboratory of Biodiversity and Evolutionary Genomics, Leuven, Belgium

^2^ Vrije Universiteit Brussel (VUB), Marine Biology Group, Brussels, Belgium

^3^ Ghent University, Marine Biology Research Group, Ghent, Belgium

^4^ Royal Belgian Institute of Natural Sciences, OD Nature, Brussels, Belgium

^5^ Université Libre de Bruxelles (ULB), Marine Biology Laboratory, Brussels, Belgium

^6^ Meise Botanic Garden, Meise, Belgium

^7^ Université de Bourgogne Franche-Comté (UBFC) UMR CNRS 6282 Biogéosciences, Dijon, France

*Correspondence: Henrik Christiansen

[henrik.christiansen@kuleuven.be](mailto:henrik.christiansen@kuleuven.be)

**Additional File 1. Samples used for reduced representation sequencing (RRS) optimization.** DNA from these samples was used for empirical restriction enzyme digestions with different enzymes (single digest *EcoRI*, *PstI*, *MspI*, or double digest *EcoRI-MspI*) and for RRS pilot libraries. Some samples were extracted twice as replicates (marked as _rep in sample ID). Three samples per species (family in the case of ostracods) were used for empirical digestions. The amphipod (*C. obesa* and *E. pontomedon*) samples and one *T. loennbergii* were used for empirical digestions, but not included in any RRS library.

| **Species** | **Sample ID** | **Origin** | **Empirical Digestion** | **RRS Library** |
| --- | --- | --- | --- | --- |
| *Macropyxis hornei* | 280 | ANT XIX-3, St. 46-7-S | *EcoRI*, *MspI* | 1 |
| *Macrocyprina rocas* | 340 | Buzios | *EcoRI*, *MspI* | 1 |
| *Macroscapha falcis* | 176 | ANT XXII-3, St. 74-6-S | *EcoRI*, *MspI* | 1 |
| *Macroscapha falcis* | 187 | ANT XXII-3, St. 74-6-E | *-* | 1 |
| *Macroscapha solecavai* | 223 | ANT XXII-3, St. 151-7-E | *-* | 1 |
| *Macroscapha falcis* | 186 | ANT XXII-3, St. 74-6-E | *-* | 1 |
| *Macroscapha opaca* | 240 | ANT XXII-3, St. 153-7-S | *-* | 1 |
| *Macroscapha solecavai* | 226 | ANT XXII-3, St. 151-7-E | *-* | 1 |
| *Macroscapha opaca* | 240_rep | ANT XXII-3, St. 153-7-S | *-* | 1 |
| *Macroscapa solecavai* | 226_rep | ANT XXII-3, St. 151-7-E | *-* | 1 |
| *Charcotia obesa* | A77 | ANTXXIX-3 PS81,  St. 162-7 | *EcoRI*, *MspI* | - |
| *Charcotia obesa* | A78 | ANTXXIX-3 PS81,  St. 162-7 | *EcoRI*, *MspI* | - |
| *Charcotia obesa* | A79 | ANTXXIX-3 PS81,  St. 162-7 | *EcoRI*, *MspI* | - |
| *Eusirus pontomedon* | HE10 | ANTXXIX-3 PS81,  St. 227-2 | *EcoRI*, *MspI* | - |
| *Eusirus pontomedon* | HE13 | ANTXXIX-3 PS81,  St. 227-2 | *EcoRI*, *MspI* | - |
| *Eusirus pontomedon* | HE14 | ANTXXIX-3 PS81,St. 227-2 | *EcoRI*, *MspI* | - |
| *Laternula elliptica* | 4C | - | *EcoRI*, *PstI*, *MspI* | 2 |
| *Laternula elliptica* | 5C | - | *EcoRI*, *PstI*, *MspI* | 2 |
| *Laternula elliptica* | 6C | - | *EcoRI*, *PstI*, *MspI* | 2 |
| *Laternula elliptica* | KGI18 | - | *-* | 2 |
| *Laternula elliptica* | KGI11 | - | *-* | 2 |
| *Laternula elliptica* | R5 | - | *-* | 2 |
| *Laternula elliptica* | R6 | - | *-* | 2 |
| *Laternula elliptica* | R7 | - | *-* | 2 |
| *Laternula elliptica* | R6_rep | - | *-* | 2 |
| *Laternula elliptica* | R7_rep | - | *-* | 2 |
| *Aequiyoldia eightsii* | 1C | - | *EcoRI*, *PstI*, *MspI* | 2 |
| *Aequiyoldia eightsii* | 2C | - | *EcoRI*, *PstI*, *MspI* | 2 |
| *Aequiyoldia eightsii* | 3C | - | *EcoRI*, *PstI*, *MspI* | 2 |
| *Aequiyoldia eightsii* | R10 | - | *-* | 2 |
| *Aequiyoldia eightsii* | R11 | - | *-* | 2 |
| *Aequiyoldia eightsii* | R12 | - | *-* | 2 |
| *Aequiyoldia eightsii* | R27 | - | *-* | 2 |
| *Aequiyoldia eightsii* | R28 | - | *-* | 2 |
| *Aequiyoldia eightsii* | KGI2 | - | *-* | 2 |
| *Aequiyoldia eightsii* | KGI5 | - | *-* | 2 |
| *Aequiyoldia eightsii* | R27_rep | - | *-* | 2 |
| *Aequiyoldia eightsii* | R28_rep | - | *-* | 2 |
| *Bathybiaster loripes* | Bat004 | Proteker II | *-* | 3 |
| *Bathybiaster loripes* | Bat062 | CEAMARC | *-* | 3 |
| *Bathybiaster loripes* | Bat076 | LASSO_ANTXXIX/3 | *EcoRI*, *PstI*, *MspI* | 3 |
| *Bathybiaster loripes* | Bat095 | ANT XXVII/3 (CAMBIO) | *-* | 3 |
| *Bathybiaster loripes* | Bat096 | ANT XXVII/3 (CAMBIO) | *EcoRI*, *PstI*, *MspI* | 3 |
| *Bathybiaster loripes* | Bat152 | ANT XXVII/3 (CAMBIO) | *EcoRI*, *PstI*, *MspI* | 3 |
| *Bathybiaster loripes* | Bat156 | JR230 | *-* | 3 |
| *Bathybiaster loripes* | Bat157 | JR275 | *-* | 3 |
| *Bathybiaster loripes* | Bat164 | POKER II | *-* | 3 |
| *Bathybiaster loripes* | Bat184 | POKER II | *-* | 3 |
| *Bathybiaster loripes* | Bat004_rep | Proteker II | *-* | 3 |
| *Bathybiaster loripes* | Bat062_rep | CEAMARC | *-* | 3 |
| *Psilaster charcoti* | Psi002 | JR15005 | *EcoRI*, *PstI*, *MspI* | 3 |
| *Psilaster charcoti* | Psi003 | JR15005 | *EcoRI*, *PstI*, *MspI* | 3 |
| *Psilaster charcoti* | Psi008 | JR15005 | *-* | 3 |
| *Psilaster charcoti* | Psi036 | REVOLTA1 | *EcoRI*, *PstI*, *MspI* | 3 |
| *Psilaster charcoti* | Psi037 | REVOLTA1 | *-* | 3 |
| *Psilaster charcoti* | Psi039 | JR275 | *-* | 3 |
| *Psilaster charcoti* | Psi040 | JR275 | *-* | 3 |
| *Psilaster charcoti* | Psi048 | CEAMARC | *-* | 3 |
| *Psilaster charcoti* | Psi063 | CEAMARC | *-* | 3 |
| *Psilaster charcoti* | Psi075 | CEAMARC | *-* | 3 |
| *Psilaster charcoti* | Psi153 | JR275 | *-* | 3 |
| *Psilaster charcoti* | Psi155 | JR275 | *-* | 3 |
| *Psilaster charcoti* | Psi164 | JR15005 | *-* | 3 |
| *Psilaster charcoti* | Psi215 | JR308 | *-* | 3 |
| *Psilaster charcoti* | Psi037_rep | REVOLTA1 | *-* | 3 |
| *Psilaster charcoti* | Psi039_rep | JR275 | *-* | 3 |
| *Trematomus bernacchii* | JRI_02 | *see* Jurajda et al. (126) | *-* | 4 |
| *Trematomus bernacchii* | JRI_03 | *see* Jurajda et al. (126) | *-* | 4 |
| *Trematomus bernacchii* | JRI_04 | *see* Jurajda et al. (126) | *-* | 4 |
| *Trematomus bernacchii* | JRI_05 | *see* Jurajda et al. (126) | *-* | 4 |
| *Trematomus bernacchii* | JRI_06 | *see* Jurajda et al. (126) | *EcoRI*, *ApeKI*, *EcoRI-MspI* | 4 |
| *Trematomus bernacchii* | JRI_07 | *see* Jurajda et al. (126) | *-* | 4 |
| *Trematomus bernacchii* | JRI_08 | *see* Jurajda et al. (126) | *EcoRI*, *ApeKI*, *EcoRI-MspI* | 4 |
| *Trematomus bernacchii* | JRI_09 | *see* Jurajda et al. (126) | *-* | 4 |
| *Trematomus bernacchii* | JRI_10 | *see* Jurajda et al. (126) | *-* | 4 |
| *Trematomus bernacchii* | JRI_11 | *see* Jurajda et al. (126) | *EcoRI*, *ApeKI*, *EcoRI-MspI* | 4 |
| *Trematomus bernacchii* | JRI_03_rep | *see* Jurajda et al. (126) | *-* | 4 |
| *Trematomus bernacchii* | JRI_04_rep | *see* Jurajda et al. (126) | *-* | 4 |
| *Trematomus loennbergii* | ROS_1352 | RSSS 2016 | *-* | 4 |
| *Trematomus loennbergii* | ROS_1353 | RSSS 2016 | *-* | 4 |
| *Trematomus loennbergii* | ROS_1354 | RSSS 2016 | *-* | 4 |
| *Trematomus loennbergii* | ROS_1417 | RSSS 2016 | *-* | 4 |
| *Trematomus loennbergii* | ROS_1418 | RSSS 2016 | *EcoRI*, *ApeKI*, *EcoRI-MspI* | 4 |
| *Trematomus loennbergii* | ROS_1419 | RSSS 2016 | *-* | 4 |
| *Trematomus loennbergii* | ROS_1420 | RSSS 2016 | *-* | 4 |
| *Trematomus loennbergii* | ROS_1421 | RSSS 2016 | *-* | 4 |
| *Trematomus loennbergii* | ROS_1484 | RSSS 2016 | *-* | 4 |
| *Trematomus loennbergii* | ROS_1485 | RSSS 2016 | *EcoRI*, *ApeKI*, *EcoRI-MspI* | 4 |
| *Trematomus loennbergii* | ROS_1487 | RSSS 2016 | *EcoRI*, *ApeKI*, *EcoRI-MspI* | - |
| *Trematomus loennbergii* | ROS_1352_rep | RSSS 2016 | *-* | 4 |
| *Trematomus loennbergii* | ROS_1353_rep | RSSS 2016 | *-* | 4 |
| *Pagodroma nivea* | 1 | BAS, Rothera Point | *-* | 5 |
| *Pagodroma nivea* | 2 | BAS, Storm Ridge | *EcoRI*, *PstI*, *MspI* | 5 |
| *Pagodroma nivea* | 3 | BAS, Storm Ridge | *EcoRI*, *PstI*, *MspI* | 5 |
| *Pagodroma nivea* | 4 | BAS, Signy Island | *EcoRI*, *PstI*, *MspI* | 5 |
| *Pagodroma nivea* | 5 | BAS, Signy Island | *EcoRI*, *PstI*, *MspI* | 5 |
| *Pagodroma nivea* | BEL-G05 | Ut 005 | *-* | 5 |
| *Pagodroma nivea* | BEL-G81 | Ta 081 | *-* | 5 |
| *Pagodroma nivea* | BEL-G20 | Pi 020 | *-* | 5 |
| *Pagodroma nivea* | BEL-G05_rep1 | Ut 005 | *-* | 5 |
| *Pagodroma nivea* | BEL-G05_rep2 | Ut 005 | *-* | 5 |
