# Supplementary material for: Facilitating population genomics of non-model organisms through optimized experimental design for reduced representation sequencing
Source: BMC Genomics. 2021 Aug 21;22:625. doi: 10.1186/s12864-021-07917-3 (PMC8380342; doi:10.1186/s12864-021-07917-3)
Supplement: Supplementary file 2 — Additional file 2. In silico estimates of the number of fragments. Estimates were produced through in silico restriction enzyme digestions for reduced representation sequencing (RRS) optimized for approximately 30× coverage. The number of fragments depends on the restriction enzyme/combination, the size window, the assumed genome size, and the reference genome used for in silico digestion. Reference genomes of related species were used as well as simulated genomes; in this case the size and GC content used to simulate the genomes are listed. The number of fragments were extrapolated to the assumed genome size. Only two different enzyme and size selection setups per target species are listed here (for RRS setups optimized for HiSeq 2500 or HiSeq 4000 sequencing runs, respectively; the same as in Table 4, Table 5, Additional File 4); further estimates can be found in spreadsheets available at https://doi.org/10.5281/zenodo.5045574. [file 12864_2021_7917_MOESM2_ESM.docx]

Supplemental Information for:

**Facilitating population genomics of non-model organisms through optimized experimental design for reduced representation sequencing**

Henrik Christiansen^1*^, Franz M. Heindler^1^, Bart Hellemans^1^, Quentin Jossart^2^, Francesca Pasotti^3^, Henri Robert^4^, Marie Verheye^4^, Bruno Danis^5^, Marc Kochzius^2^, Frederik Leliaert^3,6^, Camille Moreau^5,7^, Tasnim Patel^4^, Anton P. Van de Putte^1,4,5^, Ann Vanreusel^3^, Filip A. M. Volckaert^1^ & Isa Schön^4^

^1^ KU Leuven, Laboratory of Biodiversity and Evolutionary Genomics, Leuven, Belgium

^2^ Vrije Universiteit Brussel (VUB), Marine Biology Group, Brussels, Belgium

^3^ Ghent University, Marine Biology Research Group, Ghent, Belgium

^4^ Royal Belgian Institute of Natural Sciences, OD Nature, Brussels, Belgium

^5^ Université Libre de Bruxelles (ULB), Marine Biology Laboratory, Brussels, Belgium

^6^ Meise Botanic Garden, Meise, Belgium

^7^ Université de Bourgogne Franche-Comté (UBFC) UMR CNRS 6282 Biogéosciences, Dijon, France

*Correspondence: Henrik Christiansen

[henrik.christiansen@kuleuven.be](mailto:henrik.christiansen@kuleuven.be)

**Additional File 2. *In silico* estimates of the number of fragments.** Estimates were produced through *in silico* restriction enzyme digestions for reduced representation sequencing (RRS) optimized for approximately 30× coverage. The number of fragments depends on the restriction enzyme/combination, the size window, the assumed genome size, and the reference genome used for *in silico* digestion. Reference genomes of related species were used as well as simulated genomes; in this case the size and GC content used to simulate the genomes are listed. The number of fragments were extrapolated to the assumed genome size. Only two different enzyme and size selection setups per target species are listed here (for RRS setups optimized for HiSeq 2500 or HiSeq 4000 sequencing runs, respectively; the same as in Table 4, Table 5, Additional File 4); further estimates can be found in spreadsheets available at <https://doi.org/10.5281/zenodo.5045574>.

| Class | Target Species | Restriction Enzyme (Combination) | Size Window (bp) | Assumed Genome Size (Mb) | Reference genome (GC content) | Number of fragments |
| --- | --- | --- | --- | --- | --- | --- |
| *Ostracoda* | Macrocyprididae | *ApeKI* | 200-350 | 250 | *C. torosa* | 65,244 |
| *Ostracoda* | Macrocyprididae | *ApeKI* | 200-350 | 250 | *D. pulex* | 53,331 |
| *Ostracoda* | Macrocyprididae | *ApeKI* | 200-350 | 250 | *A. tonsa* | 15,122 |
| *Ostracoda* | Macrocyprididae | *ApeKI* | 200-350 | 250 | *P. hawaiensis* | 14,927 |
| *Ostracoda* | Macrocyprididae | *ApeKI* | 200-350 | 250 | 100 Mb (43.9) | 42,545 |
| *Ostracoda* | Macrocyprididae | *ApeKI* | 200-350 | 250 | 500 Mb (43.9) | 42,425 |
| *Ostracoda* | Macrocyprididae | *ApeKI* | 250-500 | 250 | *C. torosa* | 88,550 |
| *Ostracoda* | Macrocyprididae | *ApeKI* | 250-500 | 250 | *D. pulex* | 70,618 |
| *Ostracoda* | Macrocyprididae | *ApeKI* | 250-500 | 250 | *A. tonsa* | 22,105 |
| *Ostracoda* | Macrocyprididae | *ApeKI* | 250-500 | 250 | *P. hawaiensis* | 19,078 |
| *Ostracoda* | Macrocyprididae | *ApeKI* | 250-500 | 250 | 100 Mb (43.9) | 62,688 |
| *Ostracoda* | Macrocyprididae | *ApeKI* | 250-500 | 250 | 500 Mb (43.9) | 62,476 |
| *Malacostraca* | *Charcotia obesa* | *SbfI_MspI* | 200-330 | 27,000 | *H. azteca* | 64,094 |
| *Malacostraca* | *Charcotia obesa* | *SbfI_MspI* | 200-330 | 27,000 | *P. hawaiensis* | 25,118 |
| *Malacostraca* | *Charcotia obesa* | *SbfI_MspI* | 200-330 | 27,000 | *E. perdentatus*^†^ | 10,984 |
| *Malacostraca* | *Charcotia obesa* | *SbfI_MspI* | 200-330 | 27,000 | 10,000 Mb (38.5) | 31,590 |
| *Malacostraca* | *Charcotia obesa* | *SbfI_MspI* | 200-330 | 27,000 | 30,000 Mb (40.8) | 44,820 |
| *Malacostraca* | *Charcotia obesa* | *SbfI_MspI* | 250-450 | 27,000 | *H. azteca* | 91,927 |
| *Malacostraca* | *Charcotia obesa* | *SbfI_MspI* | 250-450 | 27,000 | *P. hawaiensis* | 41,576 |
| *Malacostraca* | *Charcotia obesa* | *SbfI_MspI* | 250-450 | 27,000 | *E. perdentatus*^†^ | 13,863 |
| *Malacostraca* | *Charcotia obesa* | *SbfI_MspI* | 250-450 | 27,000 | 10,000 Mb (38.5) | 50,760 |
| *Malacostraca* | *Charcotia obesa* | *SbfI_MspI* | 250-450 | 27,000 | 30,000 Mb (40.8) | 61,155 |
| *Malacostraca* | *Eusirus pontomedon* | *EcoRI_SphI* | 200-260 | 7,000 | *H. azteca* | 63,572 |
| *Malacostraca* | *Eusirus pontomedon* | *EcoRI_SphI* | 200-260 | 7,000 | *P. hawaiensis* | 12,800 |
| *Malacostraca* | *Eusirus pontomedon* | *EcoRI_SphI* | 200-260 | 7,000 | *E. pontomedon*^†^ | 10,986 |
| *Malacostraca* | *Eusirus pontomedon* | *EcoRI_SphI* | 200-260 | 7,000 | 10,000 Mb (38.5) | 41,580 |
| *Malacostraca* | *Eusirus pontomedon* | *EcoRI_SphI* | 200-260 | 7,000 | 30,000 Mb (40.8) | 45,325 |
| *Malacostraca* | *Eusirus pontomedon* | *EcoRI_SphI* | 250-350 | 7,000 | *H. azteca* | 101,900 |
| *Malacostraca* | *Eusirus pontomedon* | *EcoRI_SphI* | 250-350 | 7,000 | *P. hawaiensis* | 22,047 |
| *Malacostraca* | *Eusirus pontomedon* | *EcoRI_SphI* | 250-350 | 7,000 | *E. pontomedon*^†^ | 18,000 |
| *Malacostraca* | *Eusirus pontomedon* | *EcoRI_SphI* | 250-350 | 7,000 | 10,000 Mb (38.5) | 63,070 |
| *Malacostraca* | *Eusirus pontomedon* | *EcoRI_SphI* | 250-350 | 7,000 | 30,000 Mb (40.8) | 71,225 |
| *Bivalvia* | *Laternula elliptica* & *Aequiyoldia eightsii* | *ApeKI* | 200-260 | 3,000 | *C. gigas* | 69,027 |
| *Bivalvia* | *Laternula elliptica* & *Aequiyoldia eightsii* | *ApeKI* | 200-260 | 3,000 | *P. imbricata* | 64,486 |
| *Bivalvia* | *Laternula elliptica* & *Aequiyoldia eightsii* | *ApeKI* | 200-260 | 3,000 | *B. platifrons* | 53,399 |
| *Bivalvia* | *Laternula elliptica* & *Aequiyoldia eightsii* | *ApeKI* | 200-260 | 3,000 | 1,000 Mb (35.3) | 59,400 |
| *Bivalvia* | *Laternula elliptica* & *Aequiyoldia eightsii* | *ApeKI* | 200-260 | 3,000 | 5,000 Mb (34.2) | 45,480 |
| *Bivalvia* | *Laternula elliptica* & *Aequiyoldia eightsii* | *ApeKI* | 250-350 | 3,000 | *C. gigas* | 105,349 |
| *Bivalvia* | *Laternula elliptica* & *Aequiyoldia eightsii* | *ApeKI* | 250-350 | 3,000 | *P. imbricata* | 102,333 |
| *Bivalvia* | *Laternula elliptica* & *Aequiyoldia eightsii* | *ApeKI* | 250-350 | 3,000 | *B. platifrons* | 83,580 |
| *Bivalvia* | *Laternula elliptica* & *Aequiyoldia eightsii* | *ApeKI* | 250-350 | 3,000 | 1,000 Mb (35.3) | 96,600 |
| *Bivalvia* | *Laternula elliptica* & *Aequiyoldia eightsii* | *ApeKI* | 250-350 | 3,000 | 5,000 Mb (34.2) | 75,420 |
| *Asteroidea* | *Bathybiaster loripes & Psilaster charcoti* | *ApeKI* | 200-300 | 500 | *A. planci* | 76,988 |
| *Asteroidea* | *Bathybiaster loripes & Psilaster charcoti* | *ApeKI* | 200-300 | 500 | *P. miniata* | 64,466 |
| *Asteroidea* | *Bathybiaster loripes & Psilaster charcoti* | *ApeKI* | 200-300 | 500 | *P. regularis* | 62,272 |
| *Asteroidea* | *Bathybiaster loripes & Psilaster charcoti* | *ApeKI* | 200-300 | 500 | 1,000 Mb (41.3) | 42,245 |
| *Asteroidea* | *Bathybiaster loripes & Psilaster charcoti* | *ApeKI* | 200-300 | 500 | 2,000 Mb (40.4) | 36,618 |
| *Asteroidea* | *Bathybiaster loripes & Psilaster charcoti* | *ApeKI* | 250-400 | 500 | *A. planci* | 98,911 |
| *Asteroidea* | *Bathybiaster loripes & Psilaster charcoti* | *ApeKI* | 250-400 | 500 | *P. miniata* | 79,380 |
| *Asteroidea* | *Bathybiaster loripes & Psilaster charcoti* | *ApeKI* | 250-400 | 500 | *P. regularis* | 83,222 |
| *Asteroidea* | *Bathybiaster loripes & Psilaster charcoti* | *ApeKI* | 250-400 | 500 | 1,000 Mb (41.3) | 62,144 |
| *Asteroidea* | *Bathybiaster loripes & Psilaster charcoti* | *ApeKI* | 250-400 | 500 | 2,000 Mb (40.4) | 108,693 |
| *Actinopterygii* | *Trematomus bernacchii & T. loennbergii* | *EcoRI_MspI* | 200-450 | 1,500 | *N. coriiceps* | 81,605 |
| *Actinopterygii* | *Trematomus bernacchii & T. loennbergii* | *EcoRI_MspI* | 200-450 | 1,500 | 1,000 Mb (40.8) | 213,285 |
| *Actinopterygii* | *Trematomus bernacchii & T. loennbergii* | *EcoRI_MspI* | 200-450 | 1,500 | 1,800 Mb (40.8) | 214,613 |
| *Actinopterygii* | *Trematomus bernacchii & T. loennbergii* | *EcoRI_MspI* | 200-600 | 1,500 | *N. coriiceps* | 101,138 |
| *Actinopterygii* | *Trematomus bernacchii & T. loennbergii* | *EcoRI_MspI* | 200-600 | 1,500 | 1,000 Mb (40.8) | 246,555 |
| *Actinopterygii* | *Trematomus bernacchii & T. loennbergii* | *EcoRI_MspI* | 200-600 | 1,500 | 1,800 Mb (40.8) | 247,890 |
| *Aves* | *Pagodroma nivea* | *PstI* | 200-300 | 1,500 | *F. glacialis* | 66,258 |
| *Aves* | *Pagodroma nivea* | *PstI* | 200-300 | 1,500 | 1,500 Mb (41.2) | 3,270 |
| *Aves* | *Pagodroma nivea* | *PstI* | 200-300 | 1,500 | 2,000 Mb (41.2) | 3,345 |
| *Aves* | *Pagodroma nivea* | *PstI* | 250-400 | 1,500 | *F. glacialis* | 92,422 |
| *Aves* | *Pagodroma nivea* | *PstI* | 250-400 | 1,500 | 1,500 Mb (41.2) | 5,295 |
| *Aves* | *Pagodroma nivea* | *PstI* | 250-400 | 1,500 | 2,000 Mb (41.2) | 5,040 |

^†^ no reference genome of *Eusirus pontomedon* was available, instead we here used shotgun sequencing data available from a microsatellite development project for the species
