# Supplementary material for: Facilitating population genomics of non-model organisms through optimized experimental design for reduced representation sequencing
Source: BMC Genomics. 2021 Aug 21;22:625. doi: 10.1186/s12864-021-07917-3 (PMC8380342; doi:10.1186/s12864-021-07917-3)
Supplement: Supplementary file 3 — Additional file 3. Comparisons of empirical and in silico restriction enzyme digestions. Empirical Bioanalyzer results (left figure panels) with digested DNA are shown as concentration over fragment size and estimated loci numbers over locus size from in silico digestions (right figure panels) for all target taxa except fish (these are shown in Fig. 1). [file 12864_2021_7917_MOESM3_ESM.docx]

Supplemental Information for:

**Facilitating population genomics of non-model organisms through optimized experimental design for reduced representation sequencing**

Henrik Christiansen^1*^, Franz M. Heindler^1^, Bart Hellemans^1^, Quentin Jossart^2^, Francesca Pasotti^3^, Henri Robert^4^, Marie Verheye^4^, Bruno Danis^5^, Marc Kochzius^2^, Frederik Leliaert^3,6^, Camille Moreau^5,7^, Tasnim Patel^4^, Anton P. Van de Putte^1,4,5^, Ann Vanreusel^3^, Filip A. M. Volckaert^1^ & Isa Schön^4^

^1^ KU Leuven, Laboratory of Biodiversity and Evolutionary Genomics, Leuven, Belgium

^2^ Vrije Universiteit Brussel (VUB), Marine Biology Group, Brussels, Belgium

^3^ Ghent University, Marine Biology Research Group, Ghent, Belgium

^4^ Royal Belgian Institute of Natural Sciences, OD Nature, Brussels, Belgium

^5^ Université Libre de Bruxelles (ULB), Marine Biology Laboratory, Brussels, Belgium

^6^ Meise Botanic Garden, Meise, Belgium

^7^ Université de Bourgogne Franche-Comté (UBFC) UMR CNRS 6282 Biogéosciences, Dijon, France

*Correspondence: Henrik Christiansen

[henrik.christiansen@kuleuven.be](mailto:henrik.christiansen@kuleuven.be)

**Additional File 3. Comparisons of empirical and *in silico* restriction enzyme digestions.** Empirical Bioanalyzer results (left figure panels) with digested DNA are shown as concentration over fragment size and estimated loci numbers over locus size from *in silico* digestions (right figure panels) for all target taxa except fish (these are shown in Fig. 1).


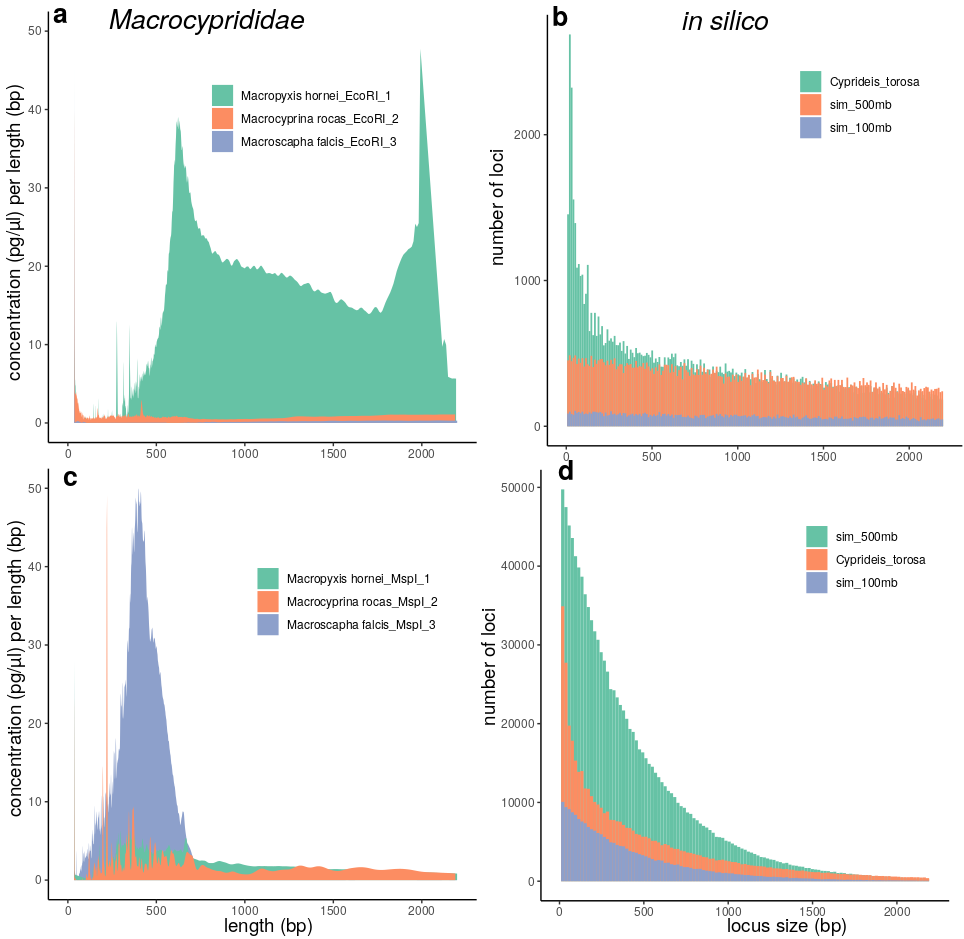


**Figure S3.1.** Empirical Bioanalyzer results with digested DNA are shown as concentration over fragment size (a, c) and estimated loci numbers over locus size from *in silico* digestions (b, d). The tests were conducted with restrictions enzymes *EcoRI* (a, b) and *MspI* (c, d). Results for the ostracod species *Macropyxis hornei*, *Macrocyprina rocas* and *Macroscapha falcis* are shown next to *in silico* estimates using a related reference genome of *Cyprideis torosa* and two simulated genomes of 100 and 500 Mb size.

**
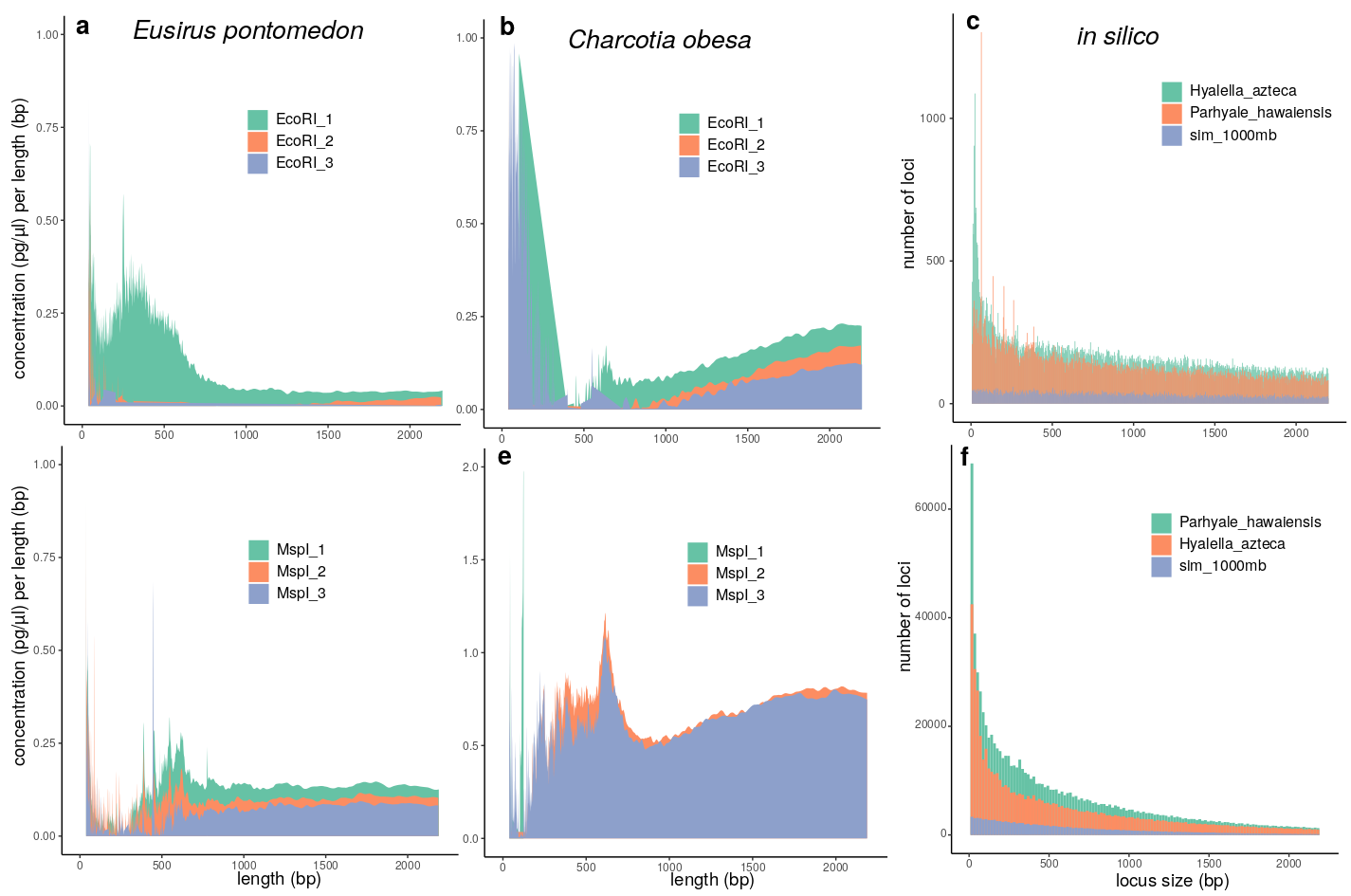
Figure S3.2.** Empirical Bioanalyzer results with digested DNA are shown as concentration over fragment size (a, b, d, e) and estimated loci numbers over locus size from *in silico* digestions (c, f). The tests were conducted with restrictions enzymes *EcoRI* (a, b, c) and *MspI* (d, e, f). Results for the amphipods species *Eusirus pontomedon* (b, e) and *Charcotia obesa* (a, d) are shown next to *in silico* estimates using reference genomes of *Hyalella azteca*, *Parhyale hawaiensis* and one simulated genome of 1000 Mb size (note that this was the absolute size used for *in silico* computations, but resulting estimates were extrapolated to 10,000 Mb).

**
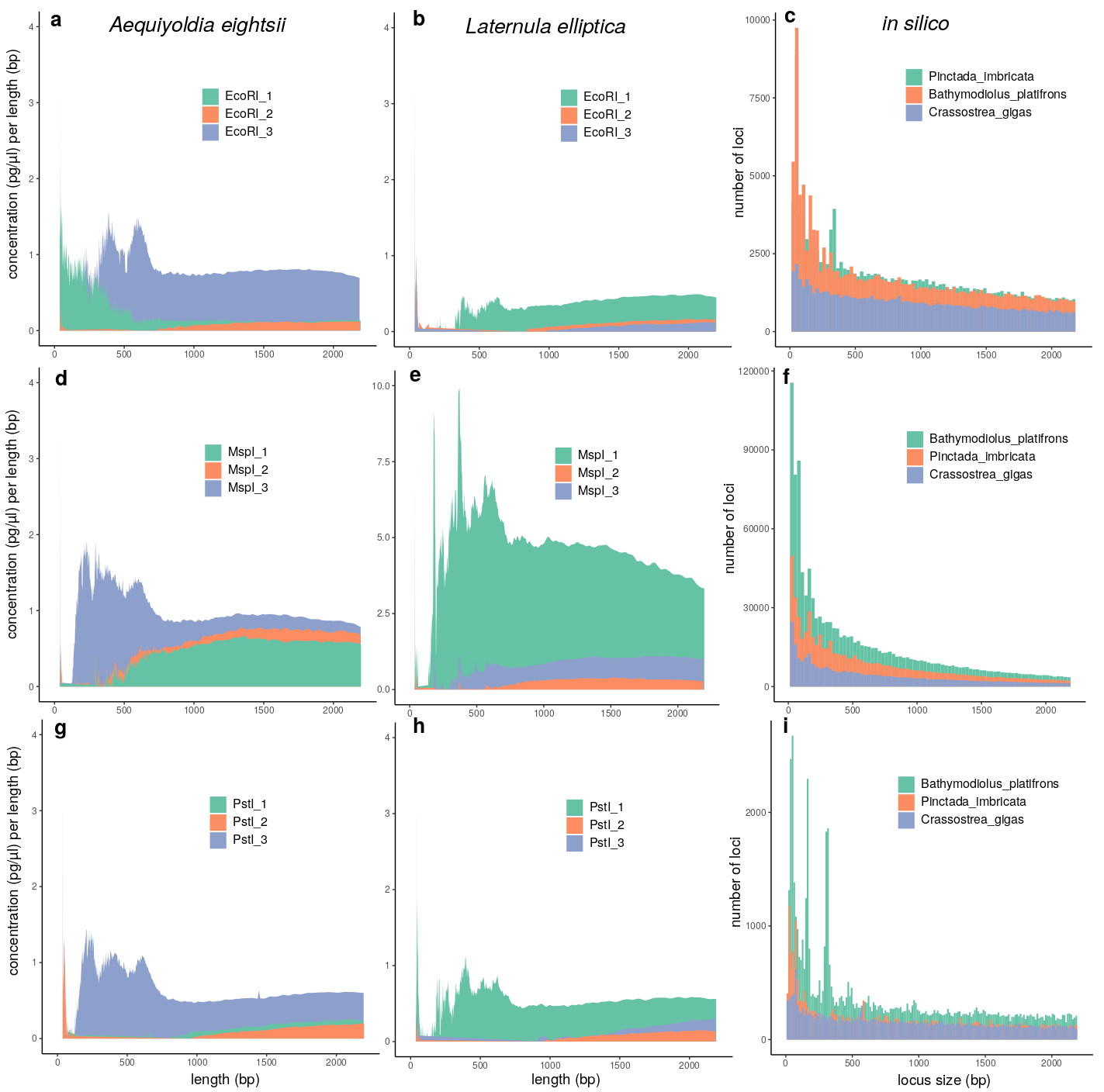
Figure S3.3.** Empirical Bioanalyzer results with digested DNA are shown as concentration over fragment size (a, b, d, e, g, h) and estimated loci numbers over locus size from *in silico* digestions (c, f, i). The tests were conducted with restrictions enzymes *EcoRI* (a, b, c), *PstI* (d, e, f) and *MspI* (g, h, i). Results for the bivalve species *Aequiyoldia eightsii* (a, d, g) and *Laternula elliptica* (b, e, h) are shown next to *in silico* estimates using reference genomes of *Bathymodiolus platifrons, Crassostrea gigas* and *Pinctada imbricata*.

**
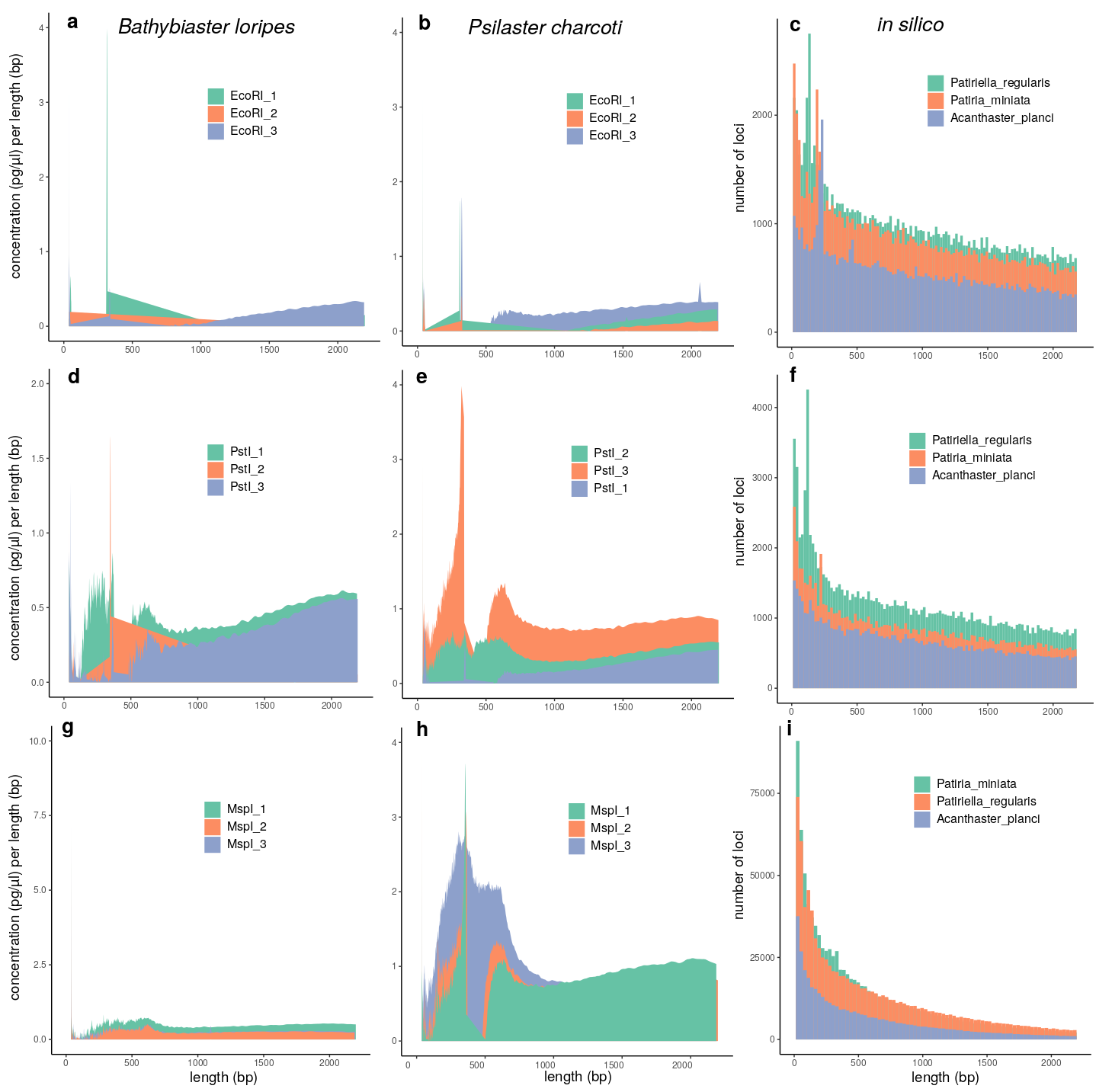
Figure S3.4.** Empirical Bioanalyzer results with digested DNA are shown as concentration over fragment size (a, b, d, e, g, h) and estimated loci numbers over locus size from *in silico* digestions (c, f, i). The tests were conducted with restrictions enzymes *EcoRI* (a, b, c), *PstI* (d, e, f) and *MspI* (g, h, i). Results for the sea star species *Bathybiaster loripes* (a, d, g) and *Psilaster charcoti* (b, e, h) are shown next to *in silico* estimates using reference genomes of *Acanthaster planci*, *Patiria miniata*, and *Patiruella regularis*.

**
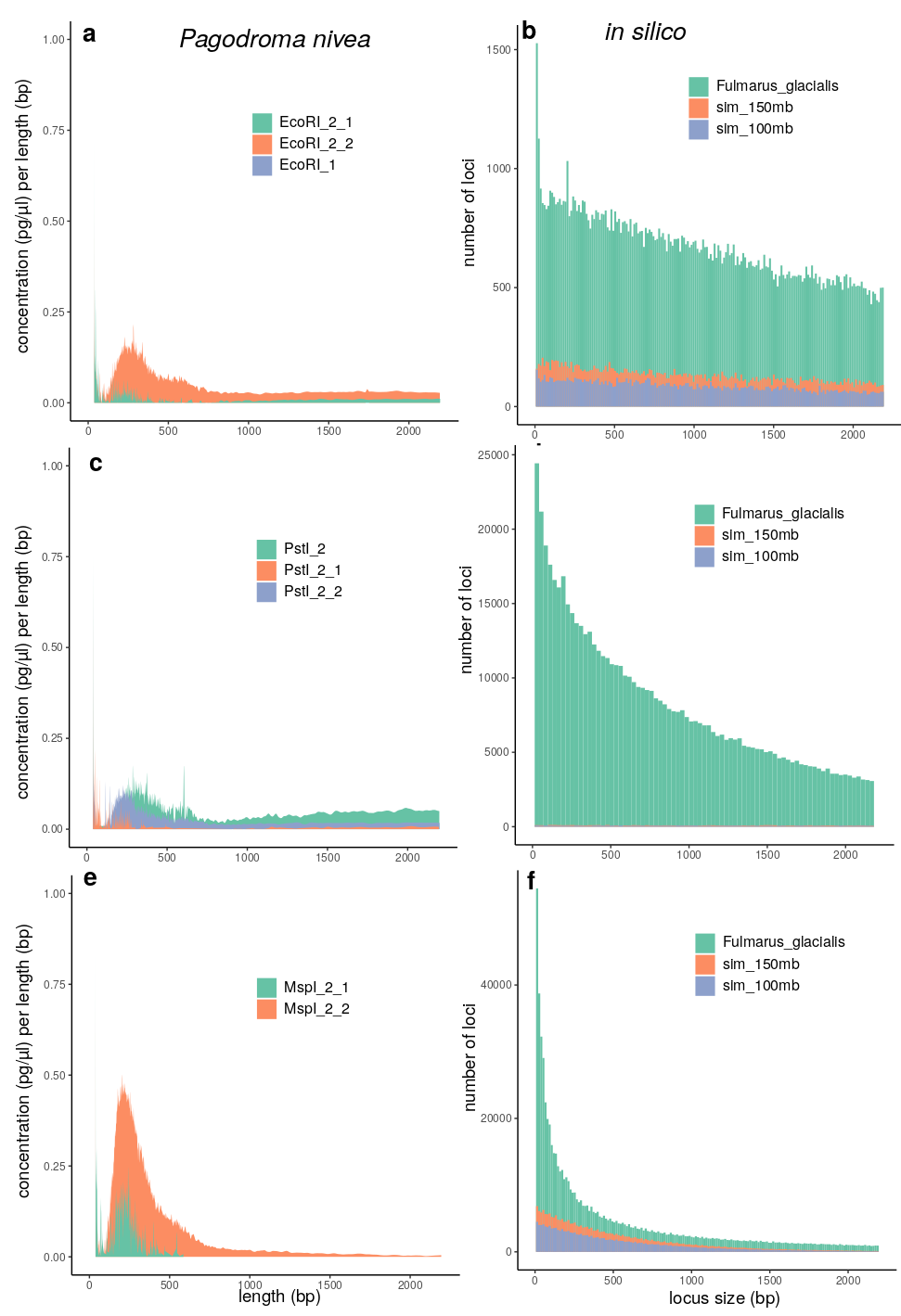
**

**Figure S3.5.** Empirical Bioanalyzer results with digested DNA are shown as concentration over fragment size (a, c, e) and estimated loci numbers over locus size from *in silico* digestions (b, d, f). The tests were conducted with restrictions enzymes *EcoRI* (a, b), *PstI* (c, d) and *MspI* (e, f). Results for the bird species *Pagodroma nivea* are shown next to *in silico* estimates using a related reference genome of *Fulmarus glacialis* and two simulated genomes of 100 and 150 Mb size (note that this was the absolute size used for *in silico* computations, but resulting estimates were extrapolated to 1000 and 1500 Mb).
