# Supplementary material for: Facilitating population genomics of non-model organisms through optimized experimental design for reduced representation sequencing
Source: BMC Genomics. 2021 Aug 21;22:625. doi: 10.1186/s12864-021-07917-3 (PMC8380342; doi:10.1186/s12864-021-07917-3)
Supplement: Supplementary file 4 — Additional file 4. Reduced representation sequencing (RRS) setups for seven individually optimized protocols. These setups were optimized in order to be run on a HiSeq 4000 platform (Illumina). The choice of restriction enzyme(s) and size window was optimized to obtain approximately 30× coverage (or half that value in a worst-case scenario) with the assumed genome size (conservatively estimated based on available information, see Table 2). Marker density was estimated as a comparable measure to the metastudy by Lowry et al. (2017) [34]. [file 12864_2021_7917_MOESM4_ESM.docx]

Supplemental Information for:

**Facilitating population genomics of non-model organisms through optimized experimental design for reduced representation sequencing**

Henrik Christiansen^1*^, Franz M. Heindler^1^, Bart Hellemans^1^, Quentin Jossart^2^, Francesca Pasotti^3^, Henri Robert^4^, Marie Verheye^4^, Bruno Danis^5^, Marc Kochzius^2^, Frederik Leliaert^3,6^, Camille Moreau^5,7^, Tasnim Patel^4^, Anton P. Van de Putte^1,4,5^, Ann Vanreusel^3^, Filip A. M. Volckaert^1^ & Isa Schön^4^

^1^ KU Leuven, Laboratory of Biodiversity and Evolutionary Genomics, Leuven, Belgium

^2^ Vrije Universiteit Brussel (VUB), Marine Biology Group, Brussels, Belgium

^3^ Ghent University, Marine Biology Research Group, Ghent, Belgium

^4^ Royal Belgian Institute of Natural Sciences, OD Nature, Brussels, Belgium

^5^ Université Libre de Bruxelles (ULB), Marine Biology Laboratory, Brussels, Belgium

^6^ Meise Botanic Garden, Meise, Belgium

^7^ Université de Bourgogne Franche-Comté (UBFC) UMR CNRS 6282 Biogéosciences, Dijon, France

*Correspondence: Henrik Christiansen

[henrik.christiansen@kuleuven.be](mailto:henrik.christiansen@kuleuven.be)

**Additional File 4. DOCX. Reduced representation sequencing (RRS) setups for seven individually optimized protocols.** These setups were optimized in order to be run on a HiSeq 4000 platform (Illumina). The choice of restriction enzyme(s) and size window was optimized to obtain approximately 30× coverage (or half that value in a worst-case scenario) with the assumed genome size (conservatively estimated based on available information, see Table 2). Marker density was estimated as a comparable measure to the metastudy by Lowry et al. (2017) (34).

| Class | Target Species | Restriction Enzyme (Combination) | Size Window (bp) | Assumed Genome Size (Mb) | Coverage^†^ | Marker Density^†^ (bp per 1 SNP) |
| --- | --- | --- | --- | --- | --- | --- |
| *Ostracoda* | Macrocyprididae | *ApeKI* | 250-500 | 250 | 35.3× | 941 |
| *Malacostraca* | *Charcotia obesa* | *SbfI_MspI* | 250-450 | 27,000 | 34.0× | 97,904 |
|  | *Eusirus pontomedon* | *EcoRI_SphI* | 250-350 | 7,000 | 30.7× | 22,898 |
| *Bivalvia* | *Laternula elliptica* and *Aequiyoldia eightsii* | *ApeKI* | 250-350 | 3,000 | 29.7 – 37.4× | 9,492 – 11,965 |
| *Asteroidea* | *Bathybiaster loripes* and *Psilaster charcoti* | *ApeKI* | 250-400 | 500 | 31.6 – 39.4× | 1,685 – 2,100 |
| *Actinopterygii* | *Trematomus bernacchii* and *T. loennbergii* | *EcoRI_MspI* | 250-600 | 1,500 | 33.3× | 4,944 |
| *Aves* | *Pagodroma nivea nivea* and *P. nivea confusa* | *PstI* | 250-400 | 1,500 | 33.8× | 5,410 |

^†^ assuming 300 million reads of 150 bp length spread over 96 individuals and 0.01 SNP/bp
