# Supplementary material for: Facilitating population genomics of non-model organisms through optimized experimental design for reduced representation sequencing
Source: BMC Genomics. 2021 Aug 21;22:625. doi: 10.1186/s12864-021-07917-3 (PMC8380342; doi:10.1186/s12864-021-07917-3)
Supplement: Supplementary file 5 — Additional file 5. Reduced representation sequencing (RRS) laboratory protocol based on the protocol from Peterson et al. (2012) [15]. The protocol is scaled for use with 192 samples and with restriction enzymes EcoRI and MspI; the reagent volumes can be scaled down/up to suit other sample numbers; if other enzymes are used, the respective reaction conditions must be adjusted. [file 12864_2021_7917_MOESM5_ESM.docx]

Supplemental Information for:

**Facilitating population genomics of non-model organisms through optimized experimental design for reduced representation sequencing**

Henrik Christiansen^1*^, Franz M. Heindler^1^, Bart Hellemans^1^, Quentin Jossart^2^, Francesca Pasotti^3^, Henri Robert^4^, Marie Verheye^4^, Bruno Danis^5^, Marc Kochzius^2^, Frederik Leliaert^3,6^, Camille Moreau^5,7^, Tasnim Patel^4^, Anton P. Van de Putte^1,4,5^, Ann Vanreusel^3^, Filip A. M. Volckaert^1^ & Isa Schön^4^

^1^ KU Leuven, Laboratory of Biodiversity and Evolutionary Genomics, Leuven, Belgium

^2^ Vrije Universiteit Brussel (VUB), Marine Biology Group, Brussels, Belgium

^3^ Ghent University, Marine Biology Research Group, Ghent, Belgium

^4^ Royal Belgian Institute of Natural Sciences, OD Nature, Brussels, Belgium

^5^ Université Libre de Bruxelles (ULB), Marine Biology Laboratory, Brussels, Belgium

^6^ Meise Botanic Garden, Meise, Belgium

^7^ Université de Bourgogne Franche-Comté (UBFC) UMR CNRS 6282 Biogéosciences, Dijon, France

*Correspondence: Henrik Christiansen

[henrik.christiansen@kuleuven.be](mailto:henrik.christiansen@kuleuven.be)

**Additional File 5. Reduced representation sequencing (RRS) laboratory protocol based on the protocol from Peterson et al. (2012) (15).** The protocol is scaled for use with 192 samples and with restriction enzymes *EcoRI* and *MspI*; the reagent volumes can be scaled down/up to suit other sample numbers; if other enzymes are used, the respective reaction conditions must be adjusted.

**Step 1.** Prepare two PCR plates with 15 µL of each DNA sample at a concentration of 20 ng/µL

**Step 2.** Restriction enzyme digestion of 192 samples with *EcoRI*-HF and *MspI* (NEB, New England Biolabs)

- Prepare master mix for 220 samples:
  - Cut Smart buffer (NEB, 2 µL per sample): 440 µL
  - *EcoRI*-HF (1 µL per sample): 220 µL
  - *MspI* (1 µL per sample): 220 µL
  - Molecular grade water (1 µL per sample): 220 µL
- Vortex master mix, spin down briefly and put on ice
- Distribute 135 µL of the mix in 8 well strip and add 5 µL to each well of the sample plates with multipipet
- Total volume in each well: 20 µL
- Incubate for **3 h at 37° C**, cool down to 10° C
- Heat inactivation: **20 min at 65° C**, cool down to 10° C

**Step 3.** Ligation (T4 DNA ligase from NEB)

- Prepare master mix for 220 samples in falcon tube
  - Cut Smart buffer (NEB, 2 µl per sample): 440 μL
  - T4 ligase (1 µl per sample): 220 μL
  - *MspI* adaptor (9 µM; 2 µl per sample): 440 µl
  - rATP (1 µl per sample): 220 µl
  - Molecular grade water (12 µl per sample): 2640 μL
- Distribute in a clean plastic tray and add 18 μL to each well containing digested DNA with multipipet
- Total volume in each well: 38 μL
- Add 2 µl *EcoRI* adaptor (0.6 µM) to each sample: **watch out, there are 8 different adaptors with 8 barcodes**. Put adaptor 1 in wells A1, A2, …till A12. Adaptor 2 in wells B1, B2, … till B12 and the same for the others
- Incubate **30 min at 22° C**, followed by **10 min at 65°C**

**Step 4.** Purification with CleanPCR beads (CleanNA; GC Biotech);

to reduce costs of CleanPCR beads, only 20 μL will be purified

- Add 20 μL beads to new plate
- Add 20 μL of digestion/ligation mixture
- Mix by carefully pipetting up and down 10 times to ensure proper mixing
- Incubate 5 min at room temperature
- Place plate on magnet for 5 min to separate beads from solution
- Remove 35 μL of the clear solution while the plate is still on the magnet. Discard solution. Avoid taking out any beads; leave ca. 5 μL of the solution behind.
- Add 200 μL of 70% ethanol and wait 30 s
- Remove 200 μL ethanol (beads are now attached much better to the wall)
- Add 200 μL of 70% ethanol, wait 30 s
- Remove all supernatant (230 μL of ethanol). Check whether all ethanol is removed. Take 10 μL multipipet to double check whether all wells are empty. Residual ethanol may interfere with downstream PCR
- Remove plate from magnet and add 40 μL elution buffer (e.g. from Qiagen kit) or pure water (Sigma)
- Mix by pipetting 10 times up and down
- Incubate 5 min
- Put plate on magnet for 5 min to separate beads from solution
- Transfer 30 μL to new plate (make sure to not transfer beads, although they are not necessarily problematic later on)

**Step 5.** PCR on individual samples

- Prepare master mix for 200 samples
  - NEB Q5 hotstart master mix (12.5 µl per sample): 2500 µl
  - Molecular grade water (8.5 µl per sample): 1700 µl
  - F-Primer (5 µM, 1 µl per sample): 200 µl
- Distribute 22 μL of the mix and add 1 μL of R-primer (5 µM): **watch out, there are 12 different primers with 12 barcodes**. Put primer 1 in wells A1, B1, … till H1. Primer 2 in wells A2, B2, … till H2 and the same for the others.
- Add 2 µl of the purified digestion-ligation mix
- Total volume: 25 μL
- Initial denaturation at **98° C for 30 s** followed by 13 cycles of **10 s at 98° C**, **30 s at 65° C** and **30 s at 72°C**. Final elongation **5 min at 72°C**.

**Step 6.** Purification with CleanPCR beads

- Purify PCR product as in step 3 but add only 20 µL of beads to 25 µL PCR product (0.8 ratio)
- Follow protocol in step 3
- The final elution volume is 25 µL and 20 µL is transferred to a new tube

**Step 7.** Quantification

- Use the Quant-iT PicoGreen protocol (Thermo Fisher Scientific Inc.) and a microplate reader or a similar photometric method to precisely quantify the individual, amplified ddRAD samples following the manufacturer’s instructions.
- If DNA quantity at this step is too low, one may try to go back to step 5 and try with more PCR cyles. This also increases the amount of PCR duplicates of course.

**Step 8.** Pooling of the samples

- Depending on the lowest concentration take 20 ng (if possible, otherwise 10 ng or 5 ng) from each sample and transfer into one single tube.
- Quantify the pooled sample with PicoGreen again and check on gel.

**Step 9.** Export to KU Leuven Genomics Core.

The library/libraries are size selected (do not forget to add adaptor length to the chosen size window) and quantified at the Genomics Core using a Pippin Prep (Sage Science) and qPCR, respectively.

See original protocol version for further details:

Peterson, B.K., Weber, J.N., Kay, E.H., Fisher, H.S., Hoekstra, H.E. (2012) Double digest RADseq: an inexpensive method for *de novo* SNP discovery and genotyping in model and non-model species. PLoS One 7(5), e37135. <https://doi.org/10.1371/journal.pone.0037135>
