# Supplementary material for: Facilitating population genomics of non-model organisms through optimized experimental design for reduced representation sequencing
Source: BMC Genomics. 2021 Aug 21;22:625. doi: 10.1186/s12864-021-07917-3 (PMC8380342; doi:10.1186/s12864-021-07917-3)
Supplement: Supplementary file 7 — Additional file 7. Results from parameter optimization for de novo assembly and genotyping. Eight parameter optimization series were conducted following Rochette & Catchen (2017) [45] to identify optimal parameters to genotype reduced representation sequencing (RRS) data with Stacks v2.4 (Rochette et al. 2019) [21]; one test series for each species/species complex. The Stacks parameter m was kept constant (m = 3), while parameters M and n were varied together from 1 to 9. Subsequently, only loci present in 80% of the samples were retained and for each M = n parameter the number of loci and polymorphic loci was plotted, as well as the proportion of these loci containing 0 to 10 or > 10 SNPs. In ostracods, the library contained DNA from a species-complex, resulting in very few shared loci across 80% of the samples. Therefore, in this case results based on loci shared by 50% of samples are shown. Optimal M = n values were decided in all cases with this information (and reported in Table 5). Note, however, that it is impossible to make absolute calls regarding the ideal value. [file 12864_2021_7917_MOESM7_ESM.docx]

Supplemental Information for:

**Facilitating population genomics of non-model organisms through optimized experimental design for reduced representation sequencing**

Henrik Christiansen^1*^, Franz M. Heindler^1^, Bart Hellemans^1^, Quentin Jossart^2^, Francesca Pasotti^3^, Henri Robert^4^, Marie Verheye^4^, Bruno Danis^5^, Marc Kochzius^2^, Frederik Leliaert^3,6^, Camille Moreau^5,7^, Tasnim Patel^4^, Anton P. Van de Putte^1,4,5^, Ann Vanreusel^3^, Filip A. M. Volckaert^1^ & Isa Schön^4^

^1^ KU Leuven, Laboratory of Biodiversity and Evolutionary Genomics, Leuven, Belgium

^2^ Vrije Universiteit Brussel (VUB), Marine Biology Group, Brussels, Belgium

^3^ Ghent University, Marine Biology Research Group, Ghent, Belgium

^4^ Royal Belgian Institute of Natural Sciences, OD Nature, Brussels, Belgium

^5^ Université Libre de Bruxelles (ULB), Marine Biology Laboratory, Brussels, Belgium

^6^ Meise Botanic Garden, Meise, Belgium

^7^ Université de Bourgogne Franche-Comté (UBFC) UMR CNRS 6282 Biogéosciences, Dijon, France

*Correspondence: Henrik Christiansen

[henrik.christiansen@kuleuven.be](mailto:henrik.christiansen@kuleuven.be)

**Additional File 7. DOCX. Results from parameter optimization for *de novo* assembly and genotyping.** Eight parameter optimization series were conducted following Rochette & Catchen (2017) (45) to identify optimal parameters to genotype reduced representation sequencing (RRS) data with Stacks v2.4 (Rochette et al. 2019) (21); one test series for each species/species complex. The Stacks parameter m was kept constant (m = 3), while parameters M and n were varied together from 1 to 9. Subsequently, only loci present in 80 % of the samples were retained and for each M=n parameter the number of loci and polymorphic loci was plotted, as well as the proportion of these loci containing 0 to 10 or >10 SNPs. In ostracods, the library contained DNA from a species-complex, resulting in very few shared loci across 80 % of the samples. Therefore, in this case results based on loci shared by 50 % of samples are shown. Optimal M=n values were decided in all cases with this information (and reported in Table 5). Note, however, that it is impossible to make absolute calls regarding the ideal value.

References

Rochette, N.C. and Catchen, J.M. (2017) Deriving genotypes from RAD-seq short-read data using Stacks. Nat. Protoc. 12, 2640–2659 <https://doi.org/10.1038/nprot.2017.123>

Rochette, N.C., Rivera-Colón, A.G. and Catchen, J.M. (2019) STACKS 2: Analytical methods for paired-end sequencing improve RADseq-based population genomics. Mol. Ecol. 28, 4737-4754 <https://doi.org/10.1111/mec.15253>


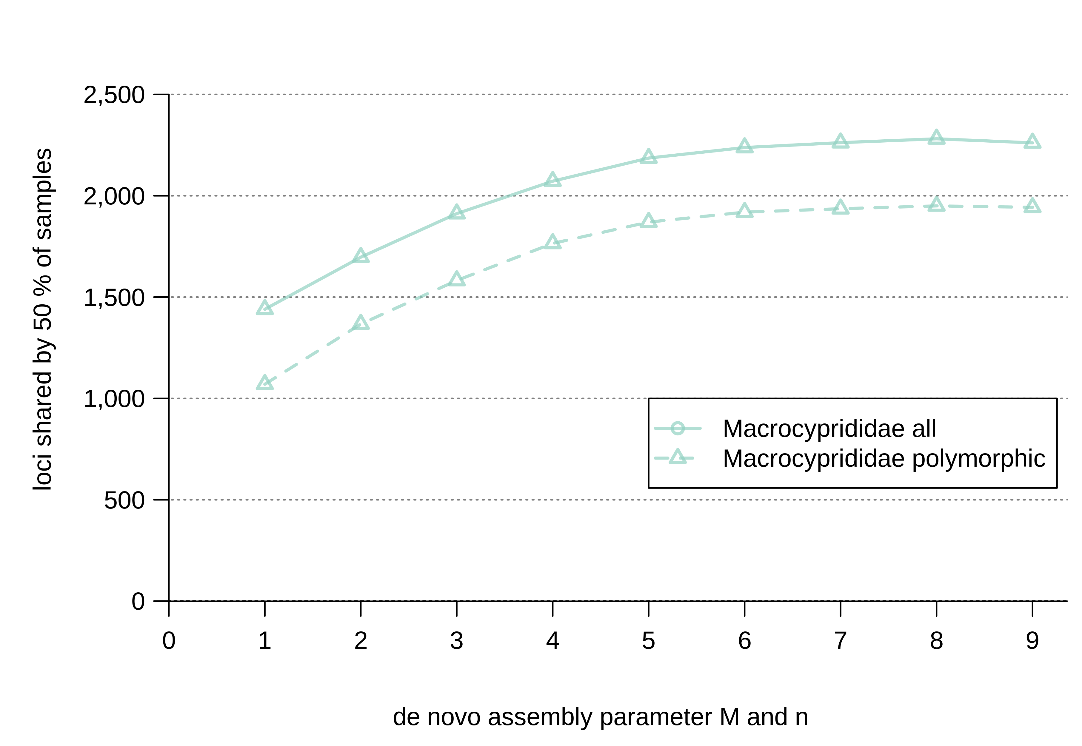

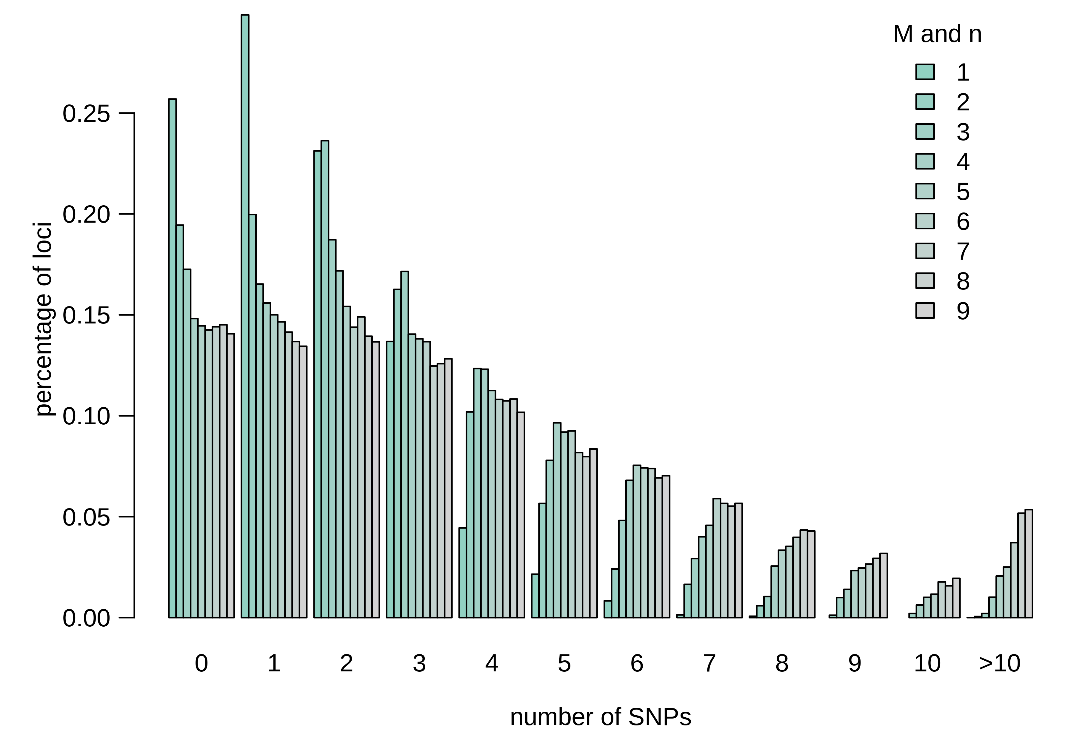


**Figure S7.1.** Number of loci and polymorphic loci shared by 50 % of samples from test library 1 across nine values for parameter M and n in Stacks v.2.4 (top) and number of SNPs per locus across the same parameter range (bottom). M = n = 6 was retained. Note that library 1 contained a species complex (*Macroscapha opaca-tensa* complex) with only few loci shared across many samples. Therefore, results of loci shared by only 50 % of the samples are shown.


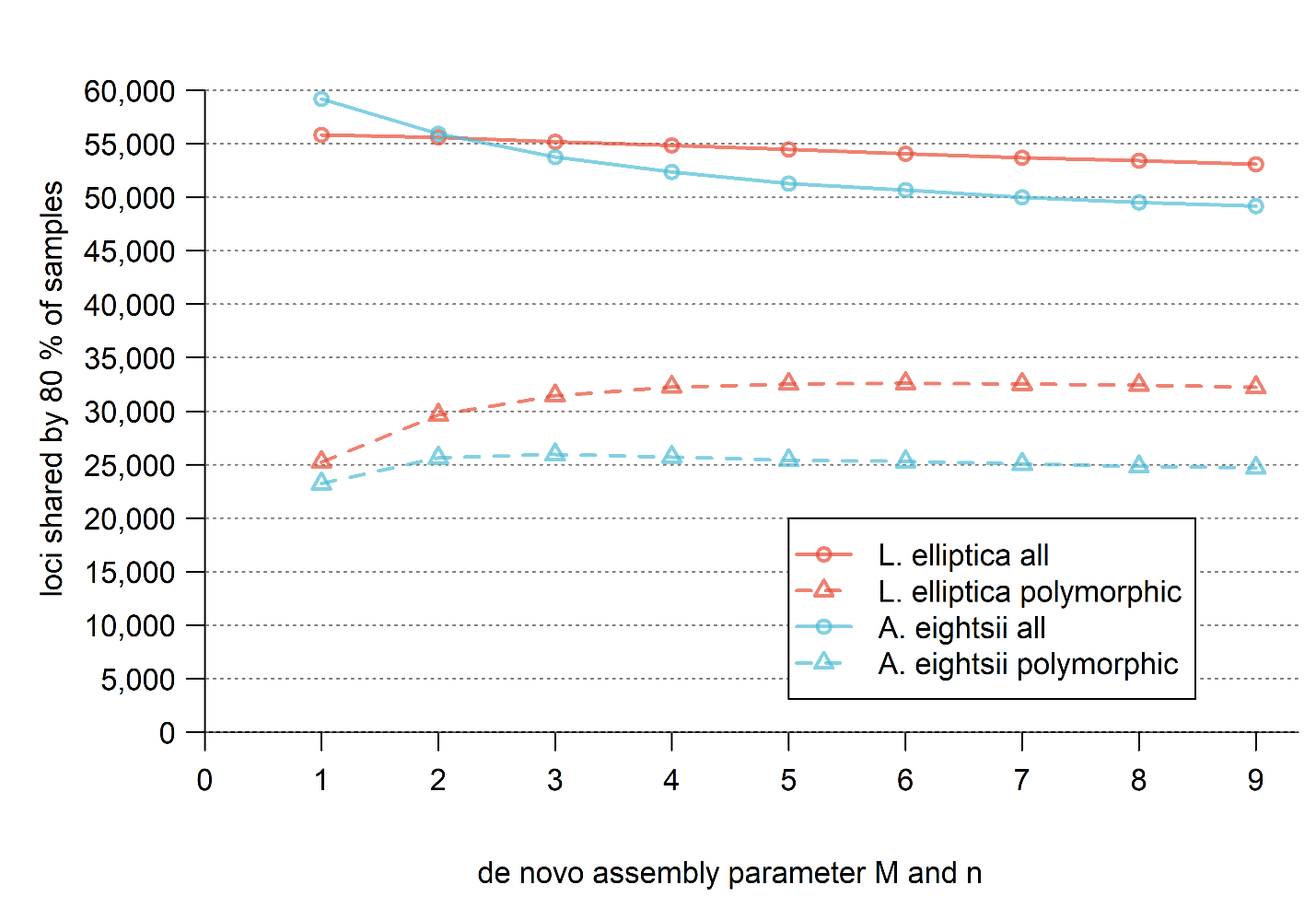


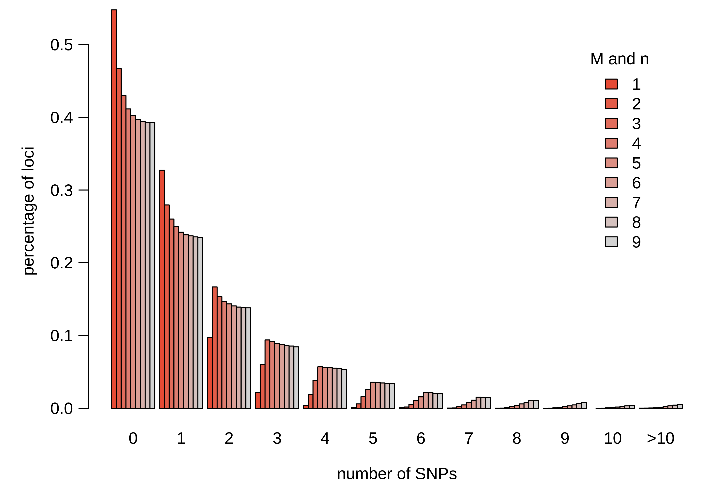

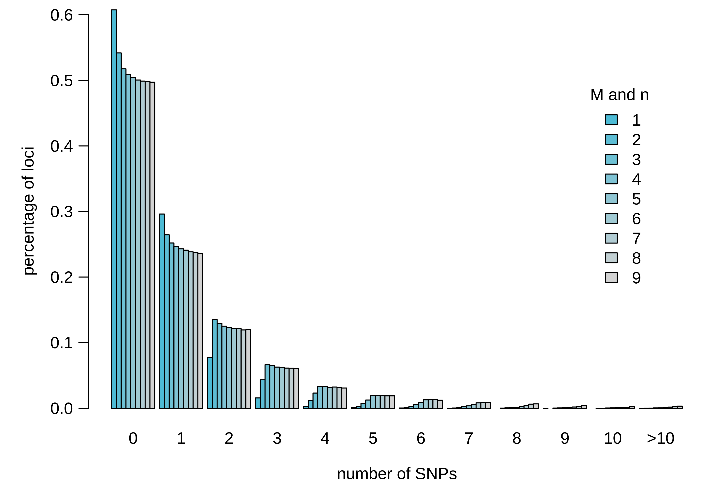


**Figure S7.2.** Number of loci and polymorphic loci shared by 80 % of samples from test library 2 across nine values for parameter M and n in Stacks v.2.4 (top) and number of SNPs per locus across the same parameter range for *Laternula elliptica* (bottom left) and *Aequiyoldia eightsii* (bottom right). M = n = 4 was retained.


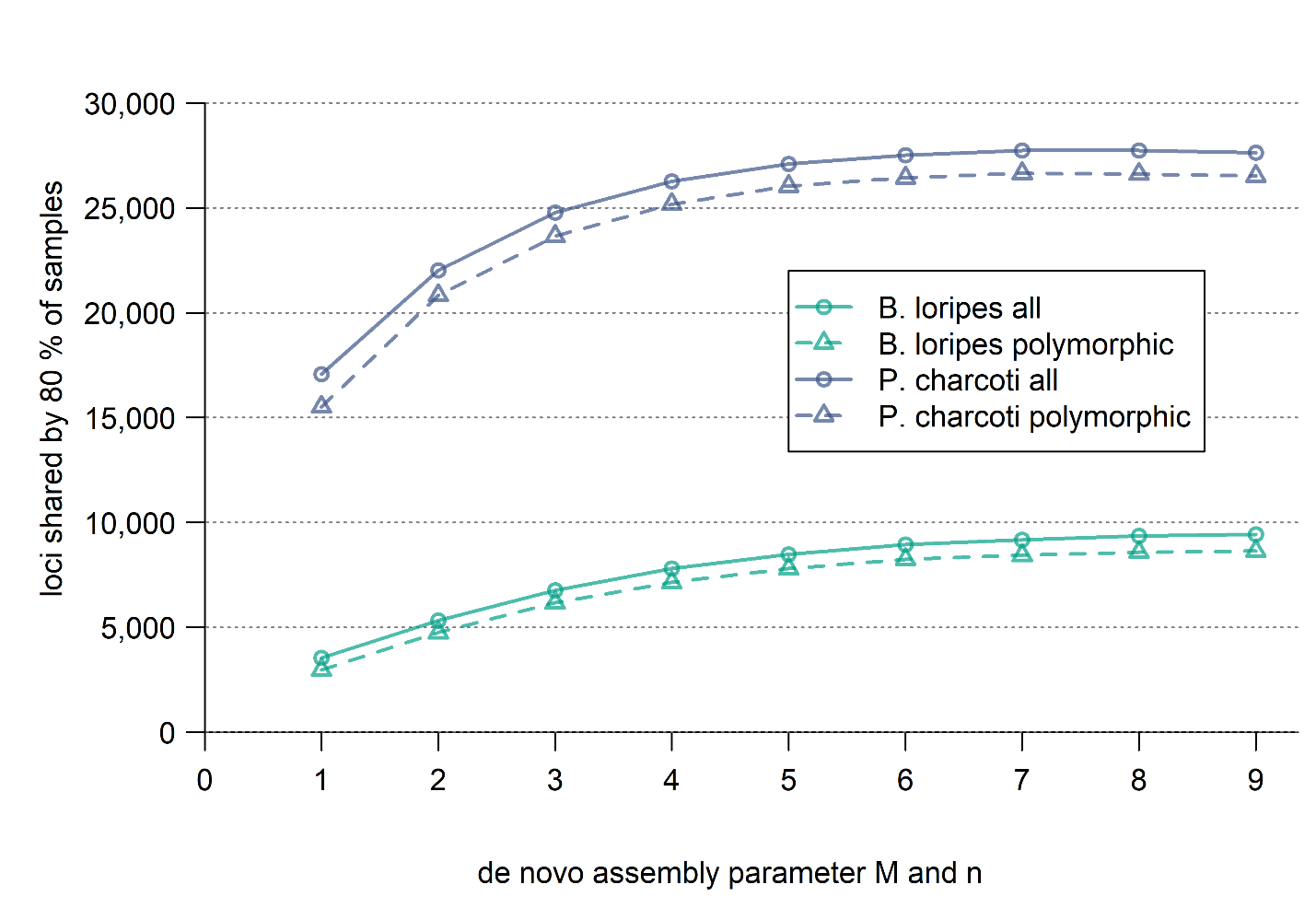


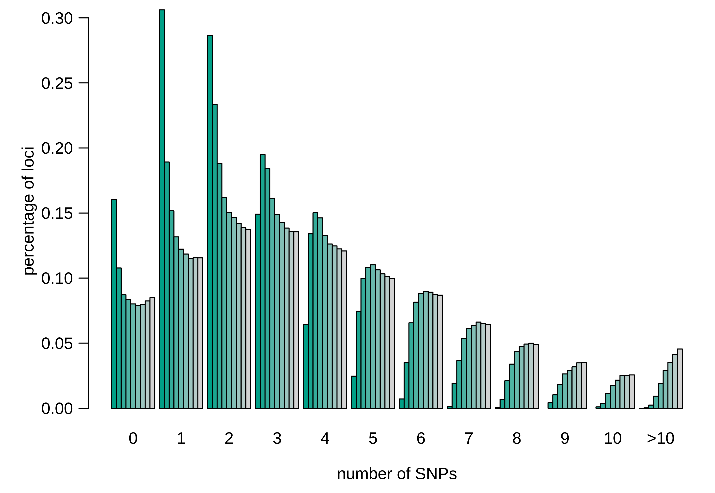

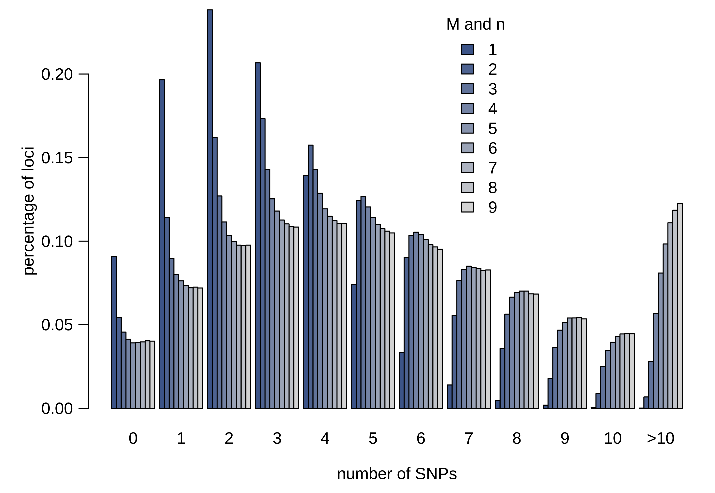


**Figure S7.3.** Number of loci and polymorphic loci shared by 80 % of samples from test library 3 across nine values for parameter M and n in Stacks v.2.4 (top) and number of SNPs per locus across the same parameter range for *Bathybiaster loripes* (bottom left) and *Psilaster charcoti* (bottom right). M = n = 5 was retained.


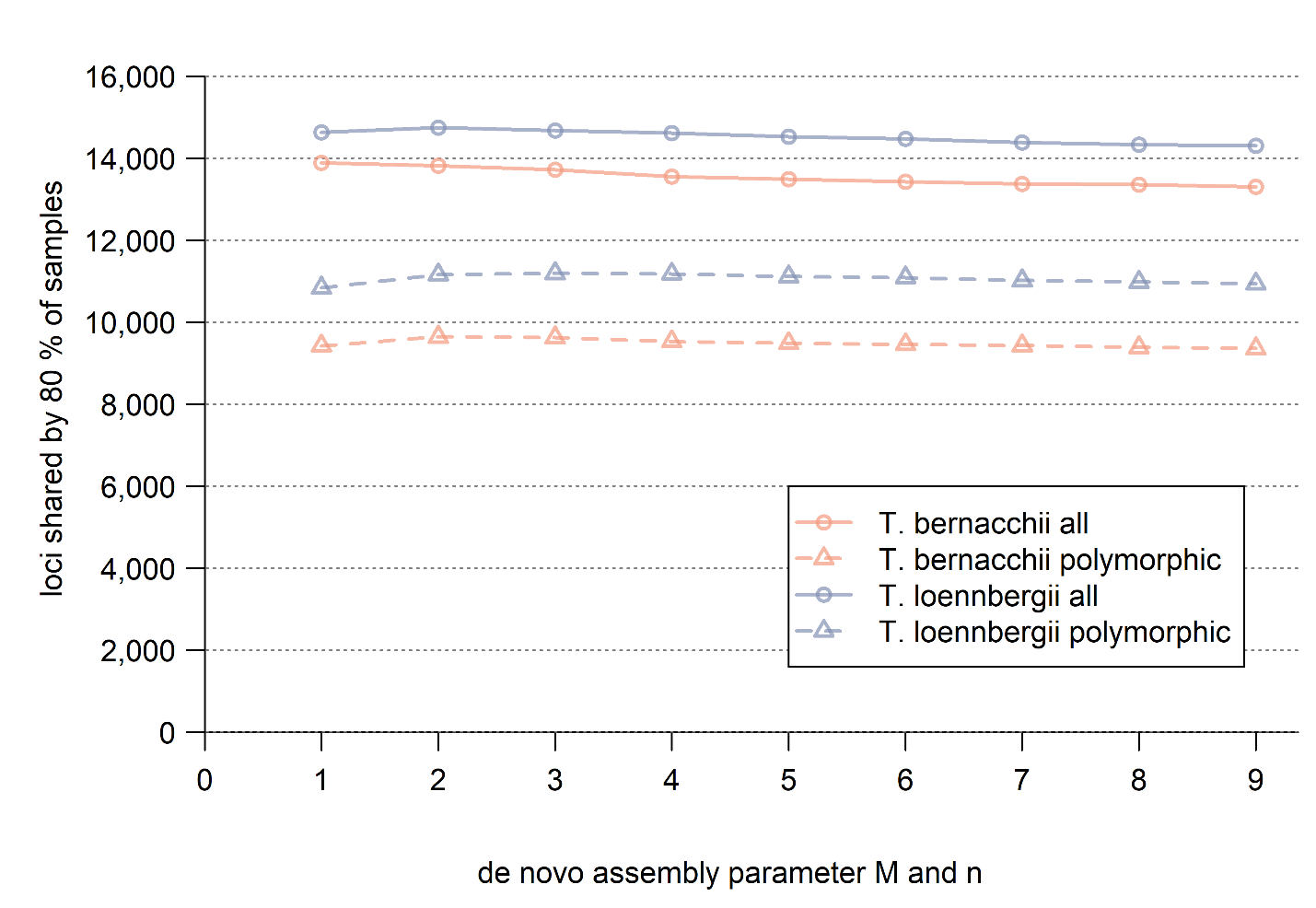


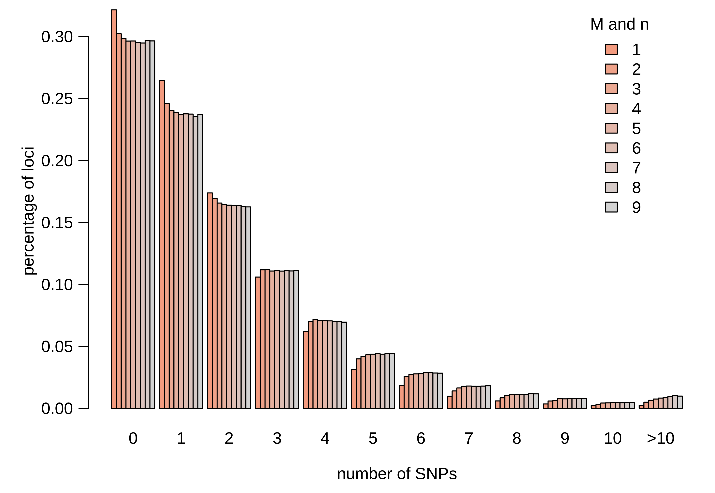

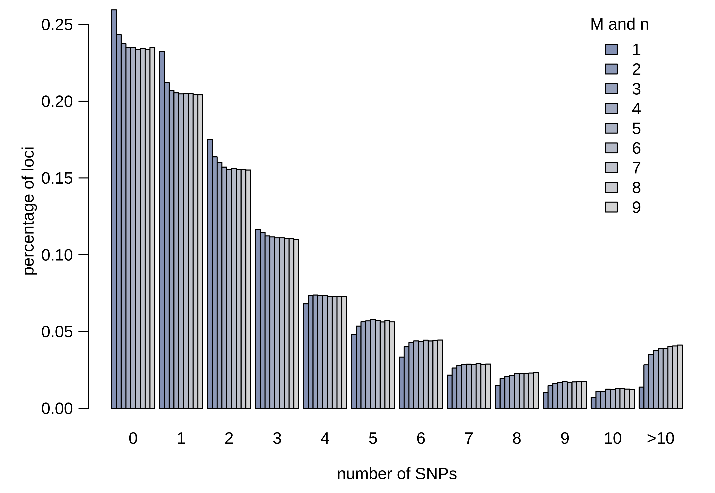


**Figure S7.4.** Number of loci and polymorphic loci shared by 80 % of samples from test library 4 across nine values for parameter M and n in Stacks v.2.4 (top) and number of SNPs per locus across the same parameter range for *Trematomus bernacchii* (bottom left) and *T. loennbergii* (bottom right). M = n = 3 was retained.


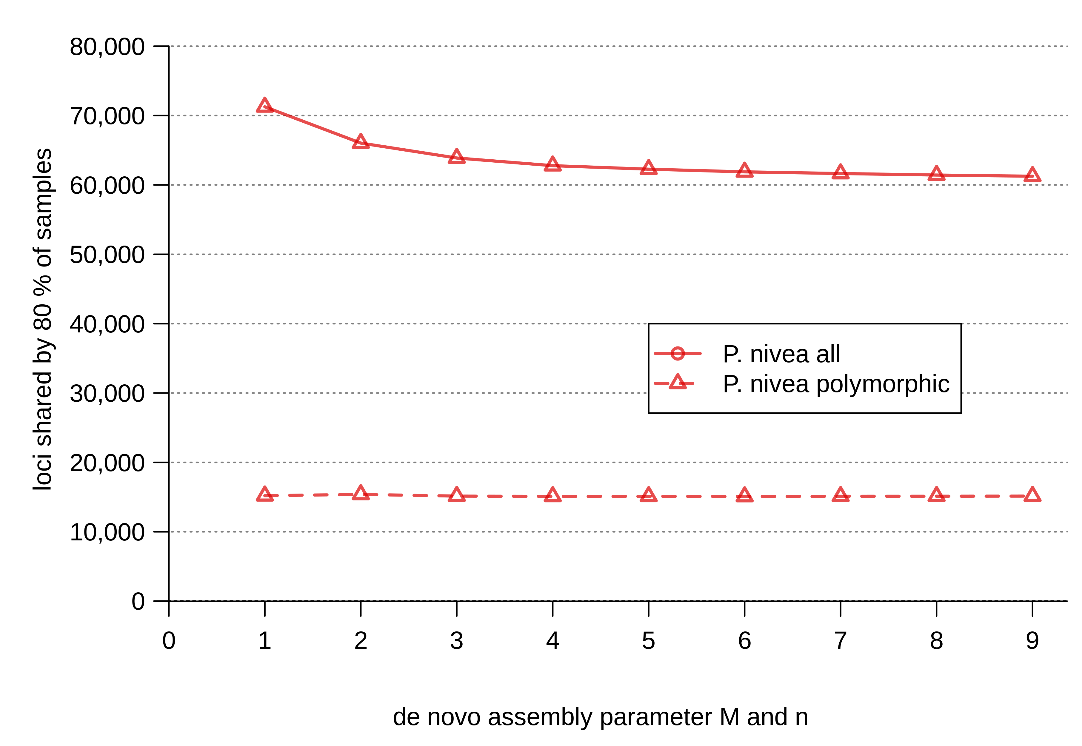

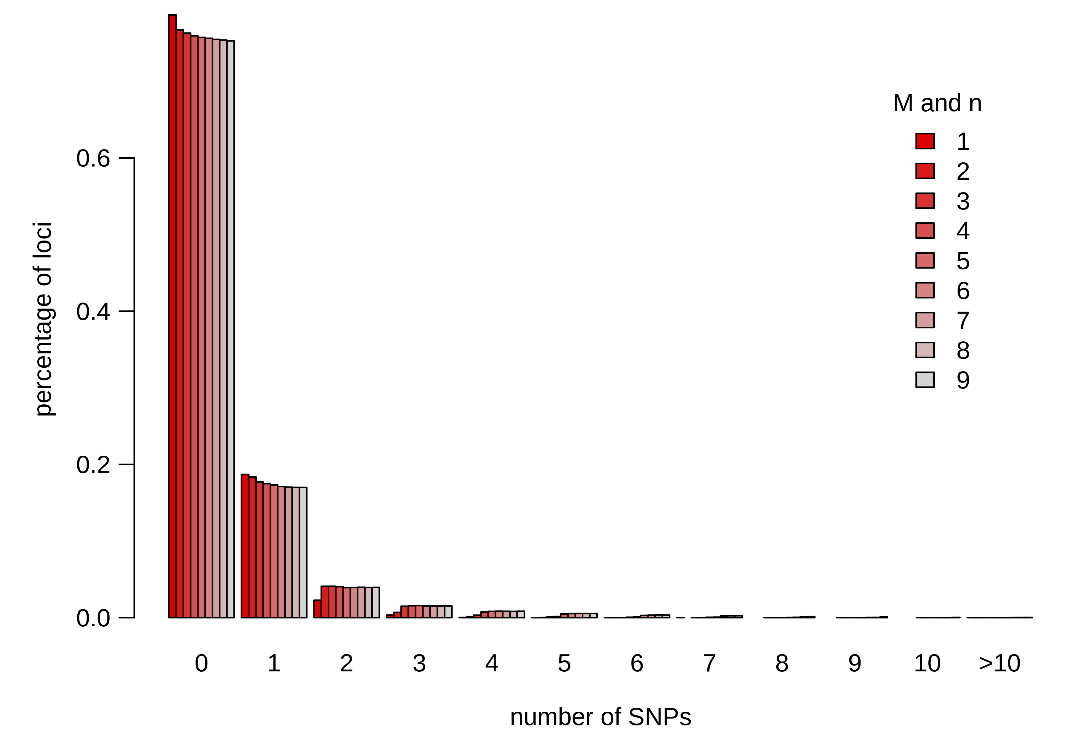


**Figure S7.5.** Number of loci and polymorphic loci shared by 50 % of samples from test library 5 across nine values for parameter M and n in Stacks v.2.4 (top) and number of SNPs per locus across the same parameter range (bottom). M = n = 3 was retained. Note that library 2 contained only few samples with likely high levels of degradation, possibly explaining the low amount of polymorphism detected.
